# Supplementary material for: Cystathionine γ-Lyase Self-Inactivates by Polysulfidation during Cystine Metabolism
Source: Int J Mol Sci. 2023 Jun 10;24(12):9982. doi: 10.3390/ijms24129982 (PMC10298322; doi:10.3390/ijms24129982)

Supplementary Information

Cystathionine γ-Lyase Self-Inactivates by Polysulfidation during Cystine Metabolism

Shoma Araki [1], Tsuyoshi Takata [1,2], Katsuhiko Ono [3], Tomohiro Sawa [3], Shingo Kasamatsu [4], Hideshi Ihara [4], Yoshito Kumagai [5], Takaaki Akaike [6], Yasuo Watanabe [1,*] and Yukihiro Tsuchiya [1,*]

1 Department of Pharmacology, Showa Pharmaceutical University, Machida 194-8543, Japan; araki@ac.shoyaku.ac.jp
2 South Bend Campus, Indiana University School of Medicine, South Bend, IN 46617, USA; ttakata@nd.edu
3 Department of Microbiology, Graduate School of Medical Sciences, Kumamoto University, Kumamoto 860-8556, Japan; onokat@kumamoto-u.ac.jp (K.O.); sawat@kumamoto-u.ac.jp (T.S.)
4 Department of Biological Chemistry, Graduate School of Science, Osaka Metropolitan University, Sakai 599-8531, Japan; kasamatsu@omu.ac.jp (S.K.); iharah@omu.ac.jp (H.I.)
5 Graduate School of Pharmaceutical Sciences, Kyusyu University, Fukuoka 812-8582, Japan; kumagai.yoshito.864@m.kyushu-u.ac.jp
6 Department of Environmental Medicine and Molecular Toxicology, Graduate School of Medicine, Tohoku University, Sendai 980-8575, Japan; takaike@med.tohoku.ac.jp
* Correspondence: yasuwata@ac.shoyaku.ac.jp (Y.W.); yatsuchi@ac.shoyaku.ac.jp (Y.T.); Tel.: +81-42-721-1549 (Y.W.); +81-42-721-1511 (ext.2121) (Y.T.)

SUPPLEMENTARY METHODS

Plasmid construction

The rat CSE mutant C69V, C83V, C108V, C136V, C171V, C205V, C207V, C251V, C254V, C255V, C306V, and C309V was subcloned into pGEX-6P vector. The nucleotide sequence of each mutant was confirmed by DNA sequencing.

SUPPLEMENTARY FIGURE LEGENDS

Figure S1. CSE β-lyase activities of recombinant CSEs. (A) CSE β-lyase activities toward β-CA (3–10 mM) of wild-type and indicated mutants were measured by coupling a color enzymatic reaction with pyruvate oxidase and peroxidase as described in the Materials and Methods section. (B) CSE β-lyase activities toward cystine (0.1-1 mM) examined for the Cys-SSH level with a sulfane sulfur-specific fluorescent probe, SSP4, as described in the Materials and Methods section. The representative data were shown of three-four experiments.

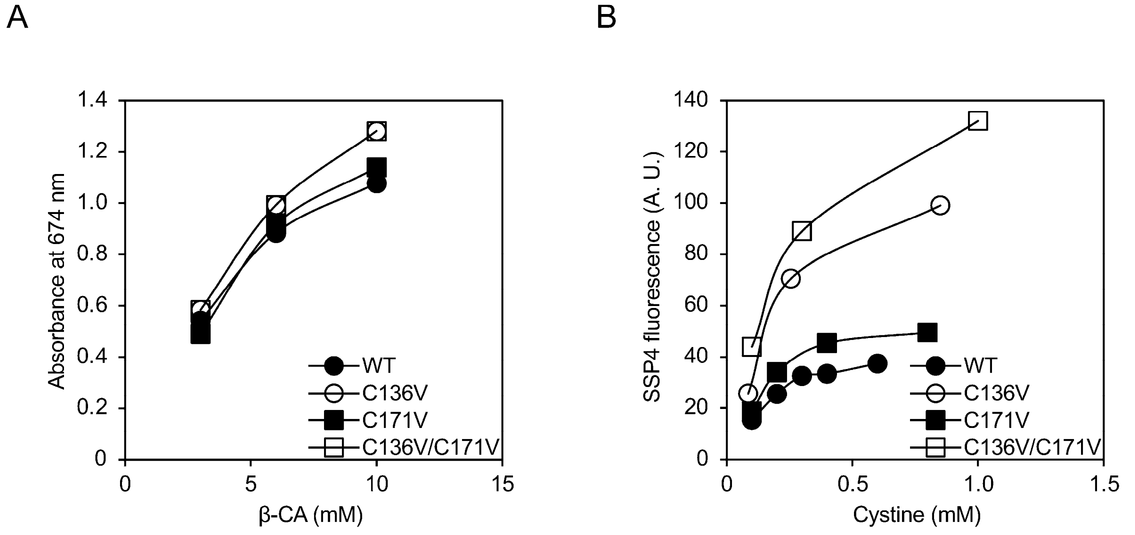

Figure S2. The cysteine residue that is sensitive to autoinactivation of CSE β-lyase activity toward cystine to generate Cys-SSH. (A) Purified recombinant CSEs preparation used in this study. Equal amounts (1 μg) of GST-cleaved wild-type CSE (WT) and the indicated mutants in E. coli cells were separated by 10% SDS-PAGE and stained by Coomassie Brilliant Blue. (B) CSE β-lyase reactions were examined for the Cys-SSH level with a sulfane sulfur-specific fluorescent probe, SSP4, as described in the Materials and Methods section. Values represent the mean ± S.E. for four

independent experiments. ****P < 0.0001 and ***P < 0.001 when compared with wild-type CSE.

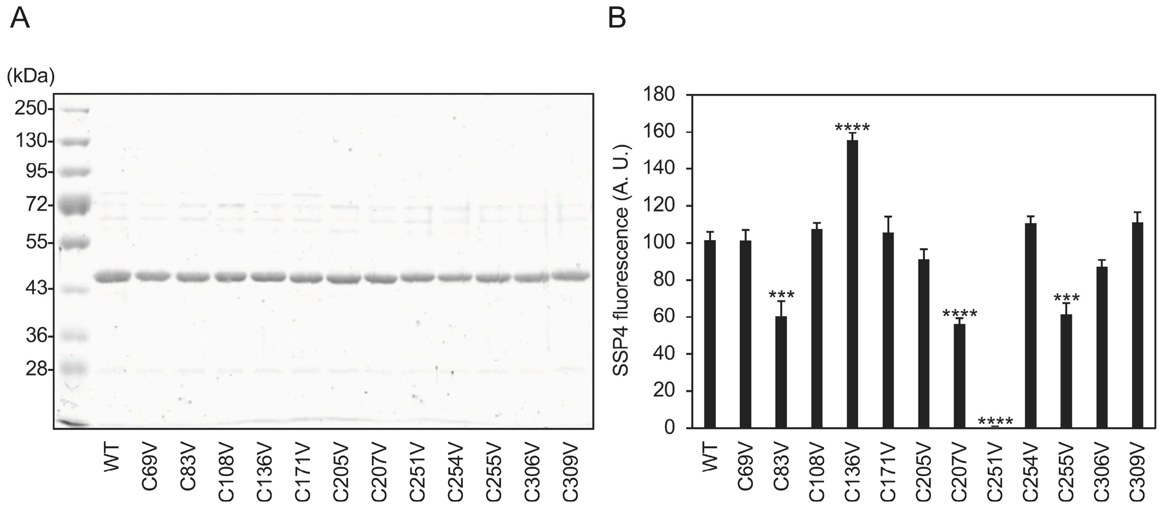

Supplement: Supplementary file 1 [file ijms-24-09982-s001.zip › ijms-2418904-supplementary.pdf]
